# Exploring Social and Assistive Domestic Robots for Older Adults: Robot Sociability, Trust, and Acceptance

**DOI:** 10.1093/geroni/igab046.1170

**Published:** 2021-12-17

**Authors:** Travis Kadylak, Megan Bayles, Wendy Rogers

**Affiliations:** 1 University of Illinois Urbana Champaign, Champaign, Illinois, United States; 2 University of Illinois Urbana-Champaign, Champaign, Illinois, United States

## Abstract

Older adults prefer to age in place, to live independently while maintaining social connection and engagement with the community. Though older adults can encounter barriers to these goals, social and assistive domestic robots hold promise for promoting independence and online/offline social engagement. However, social robots must be designed to meet their needs and preferences. Open questions remain regarding how to facilitate the development of trust and acceptance in robot support. We investigated the associations between robot social characteristics, sociability, trust, and acceptance for instrumental activities of daily living. We used an online survey to assess older adults’ perceptions towards social and assistive robots. Robots with more social abilities were rated as more acceptable and trustworthy across different task types. We discuss design implications that may promote the development of robot trust and acceptance by older adults, and ultimately help enable aging in place and social engagement.

